# Review

**Published:** 1866-09

**Authors:** 


### REVIEW.

*To the Editor of the Register:*

SIR:—There appears in the July number of the DENTAL REGISTER, an "original communication" entitled "What we most Need," wherein the writer sets forth the value of *thought* to the members of the Dental profession. My views coincide with his as far as regards the importance of *close thought;* but I do not agree with him in thinking it necessary to snub those men who have the courage to permit their names to appear in print at the end of an "original communication," "*so-called*," because their style is not as pure, or as much in accordance with the rules of syntax as it might be.

I should not, sir, however, venture to make any remarks upon the article I have alluded to were it not for one sentence, which appears towards the end. It is this, "We need a higher standard of dental literature if we expect to keep pace with the advance of science." Now, as the writer of "What we most Need" evidently looks down upon a great many who have a mistaken notion that *they* oil the *hubs* (whatever that means) of the universe, in the belief that *his* literary ability is immeasurably greater than theirs, I am tempted to examine his paper and see if the gentleman practices what he preaches.

To begin at the beginning.

We are told in the first paragraph that, "For centuries man *seemed inclined to follow the mad experiment* of living without thought." Now, without examining the philosophy of the sentence, what is the meaning of *to follow an experiment.* We hear people speak of "making an experiment," of "trying an experiment," and of "following up one experiment by another." This last cannot surely be the meaning of the phrase in the sentence quoted, for when people begin to make experiments it is a sure evidence that they *are thinking.* The fact is, as the sentence stands, it means that mankind, *by a powerful and unanimous effort of thought*, as shown in an inclination to try an experiment, was held for centuries in a divided state of mind as to the propriety of attempting an impossibility. If it does not mean that it means nothing.

In the second paragragh the writer's questions come so thick upon him that, out of breath, he answers all with another short question, regardless of whether the "hows" or the "whats" are most satisfied. In the third paragraph, second sentence, if the word "that" had been inserted before "*most*," it would save the writer from appearing to make an assertion when he intends only a quotation. The next sentence is peculiar. What purpose is "but" made to serve. Apparently it is a link between two sentences, the second of which depends upon the first. In this case, however, there is no dependence. Strip the compound sentence of its adjuncts. "Creation gives God as the author of all; but yet old thoughts may be clothed in new garbs, and beautified, etc."

When a sentence follows "but," it modifies, or should modify, the sentence preceding that word. "God is just, *but* He is merciful." We may not claim originality among men; *but* we may clothe old thoughts in new garbs. In this case, however, I am at a loss to see how the clothing of old thoughts in new garbs affects the fact that God is the author of all. The most striking feature, however, of the third paragraph is the last sentence beginning with "Not" and ending with "Plagiarist. Whether the writer was bewildered by the rapid outpouring of his own eloquence, or whether he was confused by the impudence of people who could compile from the writings of others, and send the result of their labors to the press under the title of an "original communication," I will not conjecture. I will only deal with the plain fact that here is produced in print a sentence, "so-called," without either *subject* or *predicate*.

Mr. Editor, do you think this paragraph is a fair specimen of standard Dental literature.

The second period of the next paragraph is in the same predicament as the last spoken of—subject and predicate are both absent. Perhaps this was intended for brevity; if so, it is a kind that is decidedly new to most people who speak the English language.

In the ninth paragraph it is impossible to tell whether the writer is arguing for or against the colleges.

I feel that I am trespassing, sir, too much on your valuable space; yet, one more extract, and I have done.

The first of the last three paragraphs begins thus: "We look to our journals as our hope. Let what is to be published be written with thought—close thought. Let none use *it* as an instrument to injure his brother." I have italicized the word "it," and ask what the word represents in this sentence? Certainly not "journals" nor "thought." What then? It may have been intended to represent "writing," or "the press;" but these words do not occur in any preceding portion of the paragraph. And yet only this supposition can save the unfortunate word from being a nondescript.

The fact is, sir, the writer of the article I have been quoting from, like many others, leaves too much to the imagination of the reader, and I do not think that he has attained so high a standard of dental literature that he can afford to despise the laws of syntax.

Apologizing for intruding so much on your space.

I remain, Sir,

Yours obediently,

A PUPIL.

